# Relevance of anti–platelet factor 4/heparin antibodies and platelet activation in systemic inflammatory diseases and thrombosis disorders: insight from the COVID-19 pandemic

**DOI:** 10.1016/j.rpth.2025.102701

**Published:** 2025-02-09

**Authors:** Nicolas Gendron, Dominique Helley, Johannes Thaler, Dorothée Faille, Christine Le Beller, Maxime Gruest, Jérôme Hadjadj, Aurélien Philippe, Faris Zeco, Marie Courbebaisse, Luc Darnige, Wafa Amara, Leyla Calmette, Beatrice Parfait, Claire Auditeau, Richard Chocron, Lina Khider, Laetitia Mauge, Olivier Espitia, Gérard Friedlander, Nadine Ajzenberg, David Lebeaux, Benjamin Planquette, Olivier Sanchez, Jean-Luc Diehl, COVID-HOP Study Group, Agnès Lillo-Le Louet, Benjamin Terrier, David M. Smadja

**Affiliations:** 1 Hematology department, Assistance Publique Hôpitaux de Paris, Centre-Université de Paris (APHP-CUP), Paris, France; 2 F-CRIN INNOVTE, Saint-Étienne, France; 3 Paris Cité University, INSERM, Paris Cardiovascular Research Centre, Team Endotheliopathy and Hemostasis Disorders, Paris, France; 4 Clinical Division of Haematology and Haemostaseology, Department of Medicine I, Medical University of Vienna, Vienna, Austria; 5 Paris Cité University, INSERM UMR 1144 Optimisation Thérapeutique en Neuropsychopharmacologie, Paris, France, Laboratoire d'Hématologie, AP-HP, Bichat–Claude Bernard Hospital, Paris, France; 6 Département de Pharmacovigilance, Assistance Publique Hôpitaux de Paris.Centre-Université de Paris (APHP-CUP), Paris, France; 7 Sorbonne Université, Service de Médecine interne, Hôpital Saint-Antoine, AP-HP, Imagine Institute, Laboratory for Immunogenetics of Pediatric Autoimmune Diseases, INSERM UMR 1163, Paris, France; 8 Université Paris Cité, Physiology Department, European Georges-Pompidou Hospital, APHP, INSERM U1151, Paris, France; 9 Hematology-Immunology-Transfusion Department, Hôpitaux Universitaires Paris Ile De France Ouest, Université Versailles Saint Quentin, Boulogne, France; 10 Centre de Ressources Biologiques de l'Hôpital Cochin, AP-HP.Centre-Université Paris Cité, Paris, France; 11 Paris Cité University, INSERM, Paris Cardiovascular Research Centre, F-75015 Paris, France, and Emergency department, Assistance Publique Hôpitaux de Paris-Centre (APHP-CUP), Paris, France; 12 Vascular Medicine Department, Assistance Publique Hôpitaux de Paris-Centre (APHP-CUP), Paris, France; 13 Nantes Université, CHU Nantes, Department of Internal and Vascular Medicine, l'institut du thorax, INSERM UMR1087/CNRS UMR 6291, Team III Vascular & Pulmonary diseases, Nantes, France; 14 Fondation Université Paris Cité, Paris, France; 15 Institut Pasteur, Université Paris Cité, CNRS UMR 6047, Genetics of Biofilms Laboratory, Paris, France; 16 Service de Microbiologie, Unité Mobile d’Infectiologie, Assistance Publique Hôpitaux de Paris-Centre (APHP-CUP), Hôpital Européen Georges Pompidou, Paris, France; 17 Respiratory Medicine Department, Assistance Publique - Hôpitaux de Paris-Centre (APHP-CUP), Paris, France; 18 Intensive care medicine, Assistance Publique Hôpitaux de Paris.Centre-Université de Paris (APHP-CUP), Paris, France; 19 Paris Cité University, INSERM, Paris Cardiovascular Research Centre, F-75015 Paris, France, Department of Internal Medicine, National Referral Center for Rare Systemic Autoimmune Diseases, Assistance Publique Hôpitaux de Paris-Centre (APHP-CUP), Paris, France

**Keywords:** antibodies, connectivite tissue disease, COVID-19, thrombocytopenia, vaccine, vasculitis

## Abstract

**Background:**

The increased interest in anti–platelet factor 4 (PF4)–heparin complex (anti-PF4/H) antibodies following the COVID-19 pandemic has established them as crucial players in immunothrombosis.

**Objectives:**

We aimed to investigate the involvement of anti-PF4/H antibodies during COVID-19 and after vaccination, particularly in patients with systemic inflammatory disease (SID).

**Methods:**

This retrospective study analyzed the presence of anti-PF4/H antibodies and their ability to induce platelet activation in COVID-19 patients with and without suspected heparin-induced thrombocytopenia (HIT), vaccine-induced immune thrombotic thrombocytopenia (VITT) patients, and in controls and SID patients following COVID-19 vaccination.

**Results:**

No significant increase in anti-PF4/H antibody levels was observed during COVID-19 regardless of disease severity. Despite a 2-fold increase in HIT suspicion observed during the pandemic, there was no corresponding increase in HIT diagnoses. Additionally, no significant increase in anti-PF4/H levels was noted after vaccination, even in SID patients. None of the positive anti-PF4/H antibodies detected in COVID-19 or vaccination cohorts induced platelet activation, measured by soluble P-selectin levels and flow cytometry-based on platelet microvesicle generation. Finally, in VITT patients, unlike in HIT patients, anti-PF4/H levels were strongly associated with platelet microvesicle assay and moderately with soluble P-selectin levels.

**Conclusion:**

Our study found no significant increase in anti-PF4/H antibodies in COVID-19 or after vaccination, including in SID patients. However, in VITT patients, but not in HIT patients, these antibodies were correlated with platelet activation. This finding suggests that anti-PF4/H antibodies play a different role in the pathophysiology of VITT but that their interest is limited outside clear contexts of HIT/VITT suspicion.

## Essentials

• Since the COVID-19 pandemic, interest in platelet factor 4–heparin antibodies (anti-PF4/H) has grown.
• No significant increase in anti-PF4/H levels was observed in COVID-19 patients.
• No significant increase in anti-PF4/H levels was observed after COVID-19 vaccination.
• Anti-PF4/H testing should follow current international guidelines.

## 1 Introduction

COVID-19 emerged as a pandemic associated with a strikingly high mortality rate, linked to coagulopathy and endotheliopathy [1]. This disease was associated with a high prevalence of venous thromboembolism (VTE) in patients hospitalized for severe COVID-19 [2,3]. Early in the pandemic, the International Society on Thrombosis and Haemostasis (ISTH) recommended low-molecular-weight heparin for all hospitalized COVID-19 patients [4]. The high rates of VTE in these patients, particularly those in intensive care units (ICUs), and the systematic use of low-molecular-weight heparin/heparin for prophylaxis [2,5] led to several heparin-induced thrombocytopenia (HIT) suspicions during this period [6], resulting in an increase in anti–platelet factor 4 (PF4)–heparin complex (anti-PF4/H) antibody testing [7]. Additionally, we observed a significant rise in anti-PF4/H testing, often performed alongside D-dimer assays and hemograms, due to vaccine-induced immune thrombotic thrombocytopenia (VITT) suspicions and misinformation [7].

Unlike classical HIT, these forms occur without a recent immunizing exposure to heparin [8,9]. Meanwhile, a few weeks after the initiation of mass COVID-19 vaccination campaigns around the world, several patients exhibited thrombosis and thrombocytopenia within the first month following the initial dose of the ChAdOx1 nCoV-19 vaccine (Oxford–AstraZeneca) [[10], [11], [12], [13]]. This adenovirus vector COVID-19 vaccine complication has been named VITT and is caused by anti-PF4 immunoglobulin G (IgG) mainly heparin-independent. These anti-PF4 IgGs induce platelet activation and require both therapeutic-dose anticoagulation and inhibition of FcγRIIa-mediated platelet activation by high-dose intravenous immunoglobulin [14]. ISTH guidelines for VITT laboratory diagnosis in patients presenting with clinical (thrombosis) and biological hemostasis abnormalities (thrombocytopenia) after vaccination recommend anti-PF4 testing using enzyme-linked immunosorbent assay (ELISA) followed by a functional platelet activation test in case of positive ELISA result [15]. It has been suggested that the inflammatory response induced by vaccination could trigger the production of anti-PF4/H antibodies [14], but it remains unknown if this production can also be enhanced in other inflammatory contexts such as systemic inflammatory diseases (SIDs). In this context of platelet activation, particularly in HIT, P-selectin, a glycoprotein expressed on platelets and endothelial cells that plays a role in leukocyte adhesion and platelet aggregation, emerges as an interesting biomarker of global platelet activation [16,17].

This study was designed to investigate the relevance of anti-PF4/H antibody detection according to platelet activation in COVID-19 and after COVID-19 vaccination in the general population and in patients with SIDs.

## 2 Methods

### 2.1 Patients’ cohorts

All cohorts included in this study were performed in accordance with the declaration of Helsinki.

#### 2.1.1 Patients with COVID-19

In order to investigate the prevalence and role of anti-PF4 antibodies during COVID-19, we included plasma from patients from the Hôpital Européen Georges Pompidou (HEGP, Paris, France) enrolled in the SARCODO study, previously described [18,19]. Briefly, the SARCODO study (SARCODO 2020-A01048-31A, NCT04624997) is an ongoing bicentric observational cohort study with biobank, including patients from HEGP and Cochin Hospital (Paris, France). Consecutive patients with suspected SARS-CoV-2 infection were prospectively included since March 13, 2020. Inclusion criteria were an age over 18 years old, an infectious syndrome, and a suspected COVID-19 with hospitalization criteria either in conventional wards or directly to ICU. All COVID-19-suspected patients were tested for SARS-CoV-2 infection by nasopharyngeal swabs and screened for hospitalization criteria based on local guidelines. Patients were classified according to the highest level of care they received (ward only, ward then ICU, ICU, or ICU directly). Additionally, 38 COVID-19 patients admitted to the emergency department who fulfilled the criteria for hospitalization in the ICU department were included. Among them, 15 were admitted during 1st wave of the COVID-19 pandemic (between March 13 and June 26, 2020) and 23 were admitted during 2nd wave of the COVID-19 pandemic (between June 26, 2020, and April 17, 2021). Patients were divided into two time frames, corresponding to the first day of intubation (T0), and T1 to day 7 after intubation, thus sometimes resulting in death or extubating.

#### 2.1.2 HIT-suspected patients and HIT patients

First, to investigate the incidence of HIT suspicion and HIT diagnosis during the COVID-19 pandemic, we retrospectively reviewed all consecutive patients with HIT suspicion during the COVID-19 outbreak and during the same period in 2019, from March 15 to May 15, from the RESTI-HOP study (N° 2022-03-15, CERAPHP.5, IRB registration: #00011928). The RESTI-HOP study is an ongoing study that enrolled consecutive patients with suspected HIT (mostly inpatient) referred to our HIT team in HEGP, conducted by the pharmacovigilance and hematology departments, as previously described [20]. The institutional review board of each center approved the study, and anonymous data collection was declared to the appropriate authorities. For all HIT-suspected patients included, HIT diagnosis was supported by clinical and laboratory data. Criteria for HIT suspicion were based on the 4T score [[21], [22], [23]]. An intermediate or high probability for HIT (4T score ≥4) [21] led to IgG anti-PF4/H antibodies testing by ELISA. As only a subset of anti-PF4/H antibodies is able to activate platelets and cause clinical HIT, a functional assay was required to confirm HIT diagnosis when antibody testing was positive[24]. Thus, 14C-serotonine-release assay (SRA) was performed. Furthermore, for all patients included, baseline characteristics, clinical, biological, and HIT treatment-related data were retrieved from the medical records.

Second, to investigate the differential platelet activation related to anti-PF4/H antibodies in HIT, the study also included plasma from patients diagnosed with HIT between May 2, 2018 and December 12, 2021, from the RESTI-HOP study.

#### 2.1.3 Controls and SID patients

In order to investigate the prevalence and role of anti-PF4 antibodies before and after COVID-19 vaccination, and differential platelet activation related to these antibodies, we included various cohorts. First, the study included control individuals (controls_2003–2010_) from the FARIVE case–control study conducted between 2003 and 2009 (Paris Ile-de-France Broussais 2002-11-26, FARIVE) who were free from any history of venous or arterial thrombotic disease. Exclusion criteria included a cancer diagnosis, a short life expectancy owing to other causes, and renal or liver failure. Participants’ blood samples were taken at the HEGP, Paris, France. and centrifuged at 2000 *g* for 20 minutes. Sera aliquots were snap-frozen and stored at −80°C. Sera were collected at the Biobank centralized at HEGP, Paris, France. The study was approved by the Paris Broussais–HEGP ethics committee (ethical permit: 2002-034).

Second, we included patients from the prospective COVADIS study, which enrolled patients with SID managed at Cochin Hospital, Paris, France, between January 1, 2021, and February 10, 2022, as previously described [25]. Patients with positive COVID-19 serology at baseline were excluded from the main analysis. Controls from the same hospital and during the same study period were included as controls (controls_2021_). Cases and controls received 2 doses of BNT162b2 (BioNTech/Pfizer) or ChAdOx1 nCov-19 28 days apart. Sera from SID patients and controls_2021_ were collected at specific times: day 0 (just before vaccination), day 28, and 3 months after vaccination.

Third, we included patients from the prospective and multicentric COVID-HOP study, which enrolled healthcare workers (controls_HCW_) who had received COVID-19 vaccination, as controls. Serum and plasma biobanks before and after vaccination were collected prospectively and analyzed as part of the COVID-HOP study (Clinicaltrials.gov: NCT04418375; other study identifier: APHP200609).

#### 2.1.4 Patients with thrombotic events following COVID-19 vaccination

To investigate the prevalence and role of anti-PF4 antibodies after COVID-19 vaccination in patients with thrombotic events, as well as differential platelet activation related to these antibodies, we included various cohorts. First, we included patients from the monocentric VITT study (protocol #20210917154757, CERAPHP.5, IRB registration: #00011928), which enrolled consecutive inpatients and outpatients who experienced thrombotic events within 30 days after COVID-19 vaccination (first or second dose) and who were admitted to the HEGP between March 26 and October 18, 2021, for VITT suspicion. Subsequently, 40 patients were enrolled; however, 8 were excluded due to thrombotic event after 30 days. Plasma from a total of 32 patients was included.

Second, we included 4 VITT-confirmed patients already described in the literature [[26], [27], [28]] following author agreements together with available serum samples at baseline and during follow-up.

### 2.2 Blood samples and assays

For plasma, venous blood was collected in 0.129 M trisodium citrate tubes (9NC BD Vacutainer). Platelet-poor plasma was obtained after centrifugation twice at 2500 *g* for 15 minutes, within 2 hours after sampling. Serum was obtained after centrifugation at 2500 *g* for 15 minutes. Both plasma and serum were aliquoted and stored at −80°C until testing. The detection of IgG anti-PF4/H antibodies in the patient’s plasma or serum was conducted using the IgG anti-PF4 ELISA Zymutest HIA (Hyphen Biomed), according to the manufacturer’s protocol. Patients were considered positive with an optical density (OD) ≥ 0.50; weakly positive when 0.30 ≥ OD < 0.50; or negative if OD < 0.30. The present study tested platelet activation induced by anti-PF4/H using both SRA and flow cytometry (FC) based on a platelet microvesicle assay (PMA). In both functional platelet activation tests, blood from healthy donors, free from aspirin and non-steroidal anti-inflammatory drugs for at least 10 days known to react well in the SRA and PMA was used, as previously suggested by Warkentin et al. [29].

### 2.3 SRA

A positive SRA test was defined by significant serotonin release (>30%) from donor platelets when mixed with the patient sample and low-dose heparin (0.1 U/mL), with inhibition of platelet activation by at least 50% from maximal activation values when high-dose heparin (100 U/mL) is added [20,29]. However, if the release exceeded 30% in the presence of a high unfractionated heparin (UFH) concentration, the result was considered uncertain. The result was considered negative if serotonin release was below 20% and uncertain if it was between 20% and 30% using low UFH doses with at least 3 healthy platelet donors.

### 2.4 PMA

PMA was performed as previously described [30] to detect potential activation of washed platelets from healthy volunteers induced by anti-PF4/H in patient plasma or serum. In PMA, platelet activation was challenged by Fc-receptor-blocking monoclonal antibody (anti-CD32/FcγRIIA, clone IV.3; STEMCELL Technologies) to confirm FcγRIIA-specific engagement in platelets [11,28]. Washed platelets from healthy donors were used at a platelet count of approximately 250 × 10^9^/L. Then, 20 μL of serum was incubated with the washed platelet solution (75 μL), with 5 μL of buffer alone, or UFH at 2 different final concentrations of 0.1 and 100 IU/mL. Mixtures were placed in flat-bottomed polystyrene microtiter wells and gently agitated for 1.5 hours at room temperature. After agitation, samples underwent FC analysis with Navios (Beckman-Coulter). A phycoerythrin-labeled anti-CD42b murine monoclonal antibody (SZ2, Beckman-Coulter) was used to label platelets and platelet-derived microvesicles. We observed the distribution of CD42b-positive events, which contained platelet-derived microvesicles created by activating antibodies. The left upper quadrant corresponds to activated platelets and platelet-derived microvesicles. The platelet activation index (iPA) was obtained after sample analysis with FC to quantify the extent of platelet activation and calculated as previously described [28,30]. iPA was defined as the ratio (percentage) of the number of fluorescent events in the left upper quadrant to the total number of fluorescent events in the left and right upper quadrants combined. Samples were considered positive if iPA was ≥10% at UFH 0.1 and/or 0.5 U/mL and <10% at UFH 100 U/mL, and <10% at UFH 0.1 U/mL with monoclonal antibody anti-FcγRIIA. Additional experiments were performed to sensibilize the platelet activation, with addition of PF4 (Human PF4/CXCL4 Native Protein) 10 μg/mL, as suggested by Vayne et al. [31]. Tests were realized by incubating washed platelets with either PF4 alone or PF4 + anti-FcγRIIA antibody.

### 2.5 Soluble P-selectin quantification

Soluble P-selectin (sP-sel) concentrations were quantified in platelet-poor plasma with a Human Magnetic Luminex Assay (R&D systems). Data were assessed with the Bio-Plex 200 using the Bio-Plex Manager 5.0 software (Bio-Rad).

### 2.6 Statistical analysis

Continuous data were expressed as median with IQR (25th–75th percentile) and categorical data as proportions. Continuous variables were compared using the Mann–Whitney U-test and differences in proportions were assessed with the chi-squared test for categorical variables. The association among levels of anti-PF4/H, sP-sel, and iPA and other biological parameters was assessed using a Spearman correlation test. Analyses were 2-sided, and a *P* value of <.05 was considered statistically significant. Statistical analysis was performed using GraphPad Prism 9.0 (GraphPad Software).

## 3 Results

### 3.1 Anti-PF4/H detection during COVID-19

**Figure 1:**
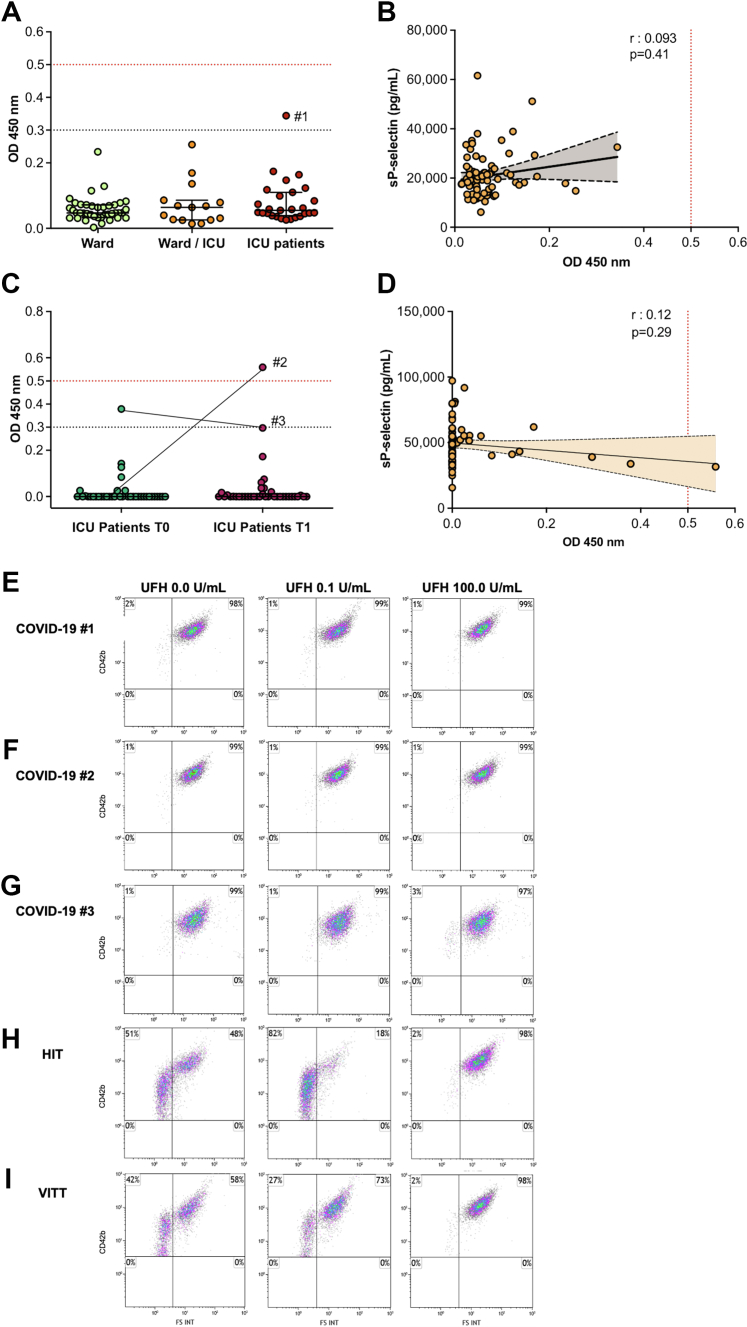
Frequency of positive immunoglobulin G (IgG) heparin–platelet factor 4 complex antibodies (anti-PF4/H) in patients with COVID-19 and association to circulating soluble P-selectin (sP-selectin). IgG anti-PF4/H (optical density; OD, 450 nm) results are considered positive with an OD > 0.50 (dashed red line: weakly positive when 0.30 < OD ≤ 0.50 (dashed black line); or negative if OD < 0.30. For platelet functional testing analysis by flow cytometry (FC), the platelet activation index, expressed as a percentage, is represented in the upper left plot of each graph.(A) IgG anti-PF4/H results in COVID-19 patients at admission, according to the highest level of care (ward only, ward then intensive care unit (ICU), ICU, or ICU directly). (B) Correlation between sP-sel and IgG anti-PF4/H levels in COVID-19 patients at admission. (C) IgG anti-PF4/H results in COVID-19 patients on the day of intubation (T0) and 7 days after intubation (T1). (D) Correlation between sP-sel and IgG anti-PF4/H levels in COVID-19 patients at T0 and T1. (E) Platelet functional testing by flow cytometry (FC) of platelet microvesicle assay (PMA) in plasma from COVID-19 patient #1 with weak positive IgG anti-PF4/H. (F) Platelet functional testing by FC of PMA in plasma from COVID-19 patient #2 with positive IgG anti-PF4/H at T1. (G) Platelet functional testing by FC of PMA in plasma from COVID-19 patient #3 with weak positive IgG anti-PF4/H at T1. (H) Platelet functional testing by FC of PMA in plasma from patient with confirmed heparin-induced thrombocytopenia (HIT) with positive IgG anti-PF4/H and a positive serotonin release assay; used as a positive control. (I) Platelet functional testing by FC of PMA in plasma from patient with confirmed vaccine-induced immune thrombotic thrombocytopenia (VITT) with positive IgG anti-PF4/H and positive serotonin release assay; used as a positive control. FS INT, forward scatter intensity; UFH, unfractionated heparin.

We first included 81 hospitalized COVID-19 patients with plasma sampling during the first 48 hours following the admission. Briefly, the median age was 66.0 years (IQR, 56.5-73.0) and 56 (69.1%) were men (Supplementary Table S1). Patients were classified according to their first referral to wards, ICU (*n* = 27) or wards (*n* = 39), then worsening in ICU (ward/ICU, *n* = 15). None of the patients exhibited high positivity results for IgG anti-PF4/H; however, 1 ICU patient (patient #1) had weakly positive result (0.34 OD, Figure 1A). No significant difference in IgG anti-PF4/H levels was observed between ward, ward/ICU, and ICU patients. Additionally, no correlation was found between plasma sP-sel circulating levels and IgG anti-PF4/H levels (Figure 1B). We next analyzed plasma from 38 ICU COVID-19 on the first day of intubation (T0) and 7 days after (T1). The median age was 66.0 years (IQR, 58.3-72.3) and 28 (73.6%) were men (Supplementary Table S2). At T0, only 1 (2.6%) patient was weakly positive for IgG anti-PF4/H (patient #2, 0.38 OD, Figure 1C). Moreover, at T1, anti-PF4/H was negative for patient #2. Only 1 (2.6%) patient who was negative at T0 turned positive at T1 (patient #3, 0.56 OD). However, there were no significant difference in IgG anti-PF4 levels or platelet counts between T0 and T1 (data not shown). Of note, after medical charts reviewing of patients #2 and #3 between T0 and T1, they did not have thrombocytopenia and/or HIT suspicion. No correlation was found between plasma sP-sel circulating levels and IgG anti-PF4/H levels (Figure 1D). Finally, we measured plasma-induced platelet-activation antibodies by PMA in all COVID-19 patients with positive anti-PF4/H results (patients #1, #2 and #3, Figure 1E–G). None of these COVID-19 patients tested showed platelet-activating properties. The iPA values with UFH at 0.0 U/mL were 2%, 1%, and 1% for patients #1, #2, and #3, respectively; iPA with UFH at 0.1 U/mL was 1% for each; and iPA with UFH at 100.0 U/mL were 1%, 1% and 3%, respectively. This is compared to a diagnosed HIT patient (Figure 1H), where iPA with UFH at 0.0 U/mL was 51%, iPA with UFH at 0.1 U/mL was 82%, and iPA with UFH at 100.0 U/mL was 2%) and a VITT patient (Figure 1I, where iPA with UFH at 0.0 U/mL was 42%, iPA with UFH at 0.1 U/mL was 27%, and iPA with UFH at 100.0 U/mL was 2%).

### 3.2 HIT suspicion during COVID-19

**Figure 2:**
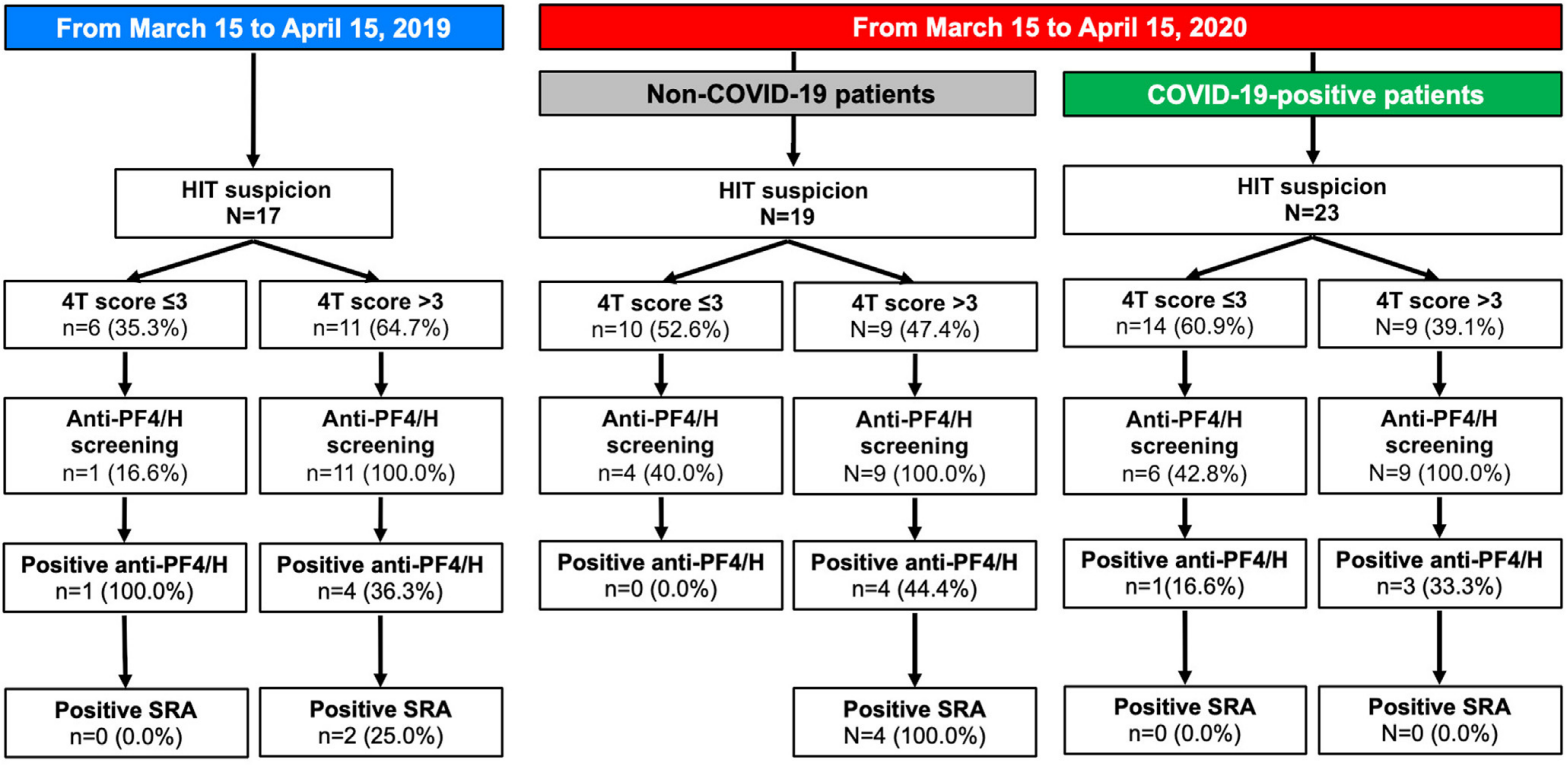
Flowchart of patients with heparin-induced thrombocytopenia (HIT) suspicion during COVID-19 outbreak and in the same period in 2019. anti-PF4/H, heparin-platelet factor 4 complex antibodies; SRA, serotonin release assay.

First, we compared the number of HIT suspicions during the first wave of the COVID-19 pandemic in France, from March 15 to April 15, 2020 (2020-study period group) and the same period in 2019. We identified, respectively; 42 and 17 consecutive HIT-suspected patients (Figure 2). Among the 2020 study period group, 23 (54.8%) were COVID-19 and 19 (45.2%) were non–COVID-19 patients. Clinical and biological characteristics were not significantly different between HIT-suspected patients from the 2019 study period group and both COVID-19 and non–COVID-19 HIT-suspected patients in the 2020 group (data not shown).

During the 2019-period study, 11 (64.7%) patients had a 4T score >3, 4 of whom (36.3%) tested positive for anti-PF4/H antibodies and 2 had a positive SRA and were confirmed with HIT diagnosis. During the 2020-period study, 9 (47.4%) non–COVID-19 and 9 (39.1%) COVID-19 patients had a 4T score >3. Among them, respectively, 4 (44.4%) and 3 (33.3%) had positive anti-PF4/H antibodies. SRA assay result was positive in the 4 non–COVID-19 tested patients but in none of COVID-19 tested patients. Results from SRA and PMA in all HIT-suspected patients with positive anti-PF4/H are provided in Supplementary Table S3.

In the 2020-study period, the only significant difference in terms of HIT suspicion criteria between COVID-19 and non-COVID-19 patients was the higher use of extracorporeal circulation circuit for cardiopulmonary bypass in non–COVID-19 patients (26.3% vs 0.0%, *P* = .03; Supplementary Table S4). Of note, there was also a trend toward a longer mean duration of heparin exposure before HIT suspicion in COVID-19 patients.

### 3.3 Anti-PF4/H detection after COVID-19 vaccination

**Figure 3:**
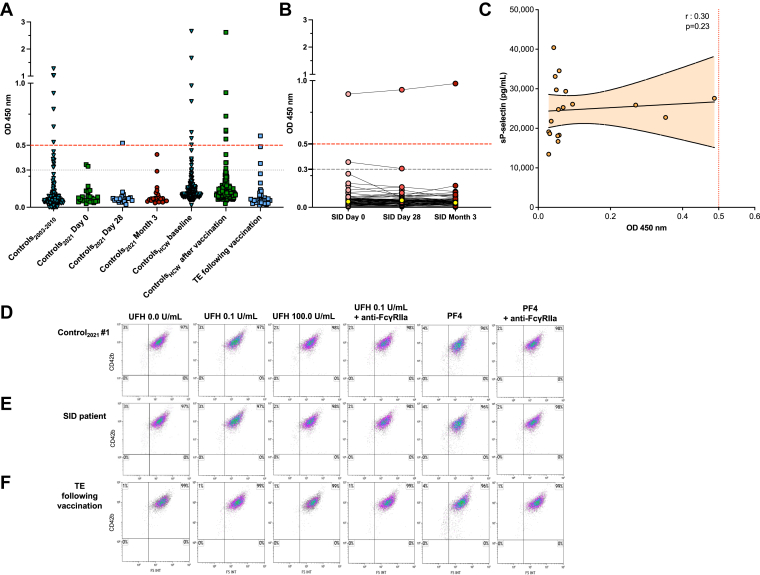
Frequency of positive immunoglobulin G (IgG) heparin–platelet factor 4 complex antibodies (anti-PF4/H) and related platelet activation from different cohorts of individuals before and after COVID-19 vaccination. Results of IgG anti-PF4/H (optical density, OD, 450 nm) are positive with an OD > 0.50 (dashed red line), weakly positive when 0.30 < OD ≤ 0.50 (dashed black line); or negative if OD < 0.30. For platelet functional testing analysis by flow cytometry (FC), the platelet activation index, expressed as a percentage, is represented in the upper left plot of each graph. (A) Frequency of positive IgG anti-PF4/H in individuals from different cohort before and after COVID-19 vaccination. (B) Frequency of positive IgG anti-PF4/H in patients with systemic inflammatory diseases (SID) before and 28 days and 3 months after COVID-19 vaccination. Yellow dots represent to patients who received their first dose of the ChAdOx1 nCoV-19 vaccine (Oxford–AstraZeneca). (C) Correlation between soluble P-selectin (sP-selectin) and IgG anti-PF4/H levels in patients evaluated after thrombotic event (TE) following COVID-19 vaccination. (D) Platelet functional testing by FC of platelet microvesicle assay (PMA) in serum of the only individual from the control_2021_ cohort with positive IgG anti-PF4/H at day 28, with unfractionated heparin (UFH) or platelet factor 4 (PF4) addition. (D) Platelet functional testing by FC of PMA in the serum from the only patient with SID with positive IgG anti-PF4/H, with UFH or PF4 addition. (E) Platelet functional testing by FC of PMA in the plasma from the only patient addressed after TE following COVID-19 vaccination and positive IgG anti-PF4/H, with UFH or PF4 addition. FS INT: forward scatter intensity; HCW, health care worker.

We measured IgG anti-PF4/H levels from samples from several cohort of patients (Table 1). First, IgG anti-PF4/H levels were assessed in sera from 122 controls_2003-2010_ patients who were never vaccinated against COVID-19 (Supplementary Table S5). Among them, 7 (5.7%) patients were positive (OD > 0.5). Then, baseline and post COVID-19 vaccination IgG anti-PF4/H levels were measured in 24 sera from controls_2021_. At baseline, all controls_2021_ were negative (Figure 3A). Two of them (8.3%) received the ChAdOx1 nCov-19 vaccine, and 22 (91.7%) received BNT162b2 (Supplementary Table S6). After COVID-19 vaccination, only 1 (4.8%) control_2021_ was positive (Control_2021_ #1, 0.52 OD) at day 28 after ChAdOx1 nCov-19 vaccine injection and none (0.0%) of the controls_2021_ were positive after 3 months.

**Table 1:** Anti–platelet factor 4 positivity in controls and anti–COVD-19 vaccination cohorts.

Sampling	Controls_2003-2010_	Controls_2021_	Controls_HCW_	Patients with SID	Sampling	Patients with TE following vaccination	VITT patients
Baseline	*N* = 122	*N* = 24	*N* = 182	*N* = 61	At VITT suspicion	*N* = 32	*N* = 4
Positive, *n* (%)	7 (5.7)	0 (0.0)	7 (3.8)	1 (1.6)			
Negative, *n* (%)	115 (94.3)	24 (100.0)	175 (96.2)	60 (98.4)			
Day 28	NA	*N* = 21	*N* = 21	*N* = 60	Positive – *n* (%)	1 (3.1)	4 (100.0)
Positive, *n* (%)		1 (4.8)	1 (4.8)	1 (1.7)			
Negative, *n* (%)		20 (95.2)	20 (95.2)	59 (98.3)	Negative – *n* (%)	31 (96.9)	
Month 3	NA	*N* = 24	*N* = 159	*N* = 49			
Positive, *n* (%)		0 (0.0)	5 (3.1)	1 (2.0)			
Negative, *n* (%)		24 (100.0)	154 (96.9)	48 (98)			

The presence of immunoglobulin G anti–platelet factor 4 in plasma or serum was evaluated by Zymutest HIA (samples positive if optical density, OD, > 0.50).

HCW, health care worker from dentification de marqueurs associés au risque d’infection par le SARS-CoV2 et au statut COVID+ asymptomatique versus COVID+ symptomatique grâce à la constitution d’unecollection biologique au cours du dépistage sérologique SARS-CoV-2 chez les professionnels de l’APHP (COVID-HOP) study; SID, systemic inflammatory disease; TE, thrombotic event; VITT, vaccine-induced thrombosis thrombocytopenia; NA: nonavailable.

In the cohort of 182 controls_HCW_ (Supplementary Table S7), we measured IgG anti-PF4/H levels in sera at baseline and after COVID-19 vaccination. At baseline, 7 (3.8%) of controls_HCW_ were positive. Sera from 75 controls_HCW_ were analyzed after the first vaccine injection and 106 after 2 injections. Among all, 37 (20.4%) received at least 1 dose of ChAdOx1 nCov-19 vaccine. Furthermore 21 and 159 controls_HCW_ were sampled, respectively, after day 28 and 3 months following vaccination. Among controls_HCW_, 1 (4.8%) was positive after day 28 and 5 (3.1%) were positive after 3 months.

We also measured IgG anti-PF4/H levels in the sera from 61 patients with SID at baseline and after COVID-19 vaccination (Figure 3B). Baseline characteristics of SID patients are described in Table 2. The median age of patients with SID was 52.0 years (IQR, 36.5-67.5) with a majority of women (65.6%). Among them, the most common diagnoses included anti-neutrophil cytoplasmic antibody-associated vasculitis (24.6%), systemic lupus erythematosus (18.0%), and systemic sclerosis (13.1%), and 23% of patients had an active disease at the time of vaccination. Only 1 (1.6) patient received ChAdOx1 nCov-19 vaccination and all others (98.4%) received BNT162b2 vaccination. Only 1 patient with SID tested positive at baseline (1.6%) and remained positive at both day 28 (1.7%) and after 3 months (2.0%) following vaccination with BNT162b2. None of them exhibited thrombocytopenia or thrombosis.

**Table 2:** Demography and clinical characteristics of patients with systemic inflammatory disease at vaccination (***n* = 61**).

Clinical characteristics	
Women, *n* (%)	40 (65.6)
Age, (y), median (IQR)	52.0 (36.5-67.5)
Diagnosis, *n* (%)	
Vasculitis	
Anti-neutrophil cytoplasmic antibody-associated vasculitis	15 (24.6)
Behçet’s	3 (4.9)
Cryoglobulinemia vasculitis	2 (3.3)
Large vessel vasculitis	4 (6.6)
Connective tissue disease	
Systemic lupus erythematosus	11 (18.0)
Systemic sclerosis	8 (13.1)
Sjogren syndrome	2 (3.3)
Myositis	5 (8.2)
Inflammatory rheumatic diseases	
Rheumatoid arthritis	3 (4.9)
Spondyloarthritis	1 (1.6)
Sarcoidosis	3 (4.9)
Others	4 (6.6)
Disease activity status, *n* (%)	
Active disease	14 (23.0)
Renal involvement	15 (24.6)
COVID-19 vaccine, *n* (%)	
BNT162b2	60 (98.4)
ChAdOx1 nCoV-19	1 (1.6)

**Figure 4:**
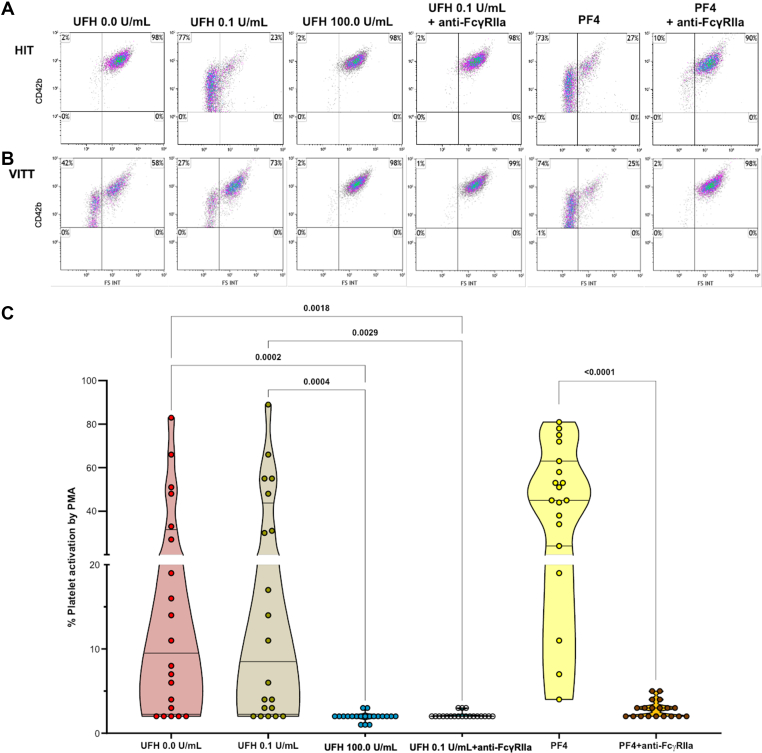
Platelet activation in confirmed heparin-induced thrombocytopenia (HIT) and confirmed vaccine-induced immune thrombotic thrombocytopenia (VITT) with heparin or platelet factor 4 addition. For platelet functional testing analysis by flow cytometry (FC), the platelet activation index, expressed as a percentage, is represented in the upper left plot of each graph. (A) Platelet functional testing by FC of platelet microvesicle assay (PMA) in the plasma from a patient with confirmed HIT with positive immunoglobulin G heparin–platelet factor 4 (PF4) complex antibodies and positive serotonin release assay. (B) Platelet functional testing by FC of PMA in the serum from a patient with confirmed VITT with positive immunoglobulin G immunoglobulin G heparin-PF4 complex antibodies and positive serotonin release assay; used as a positive control. (C)Truncated violin plots showing the index of platelet activation by PMA in 20 serum samples from 4 confirmed VITT patients at diagnosis and during follow-up. FS INT: forward scatter intensity; UFH, unfractionated heparin.

We next measured the presence of IgG anti-PF4/H antibodies in plasma from consecutive in- and outpatients addressed for thrombotic event within 30 days after vaccine injection. In total, 32 patients were included, with a median age of 61 years (IQR, 47.0-72.8) and 50% were men. Administered vaccines included BNT162b2, ChAdOx1 nCov-19, and mRNA-1273 which were given to 19 (61.3%), 11 (35.5%), and 1 (3.2%) patient, respectively (Table 3). Among them, 26 (81.3%) had VTE and 6 (18.8%) had arterial thrombosis. The median time between vaccination and thrombotic event or laboratory measurement was 11.0 (IQR, 6.3-19.8) and 17.5 days (IQR, 10.0-38.5), respectively. None of the patients had thrombocytopenia and only 1 (3.1%) patient was positive for IgG anti-PF4/H at 0.58 OD. In this cohort of patients with thrombosis following COVID-19 vaccination, no correlation was found between plasma sP-sel circulating levels and IgG anti-PF4/H levels (Figure 3C). Finally, platelet-activating antibodies were measured by FC based on PMA in all controls and patients previously described with anti-PF4/H positive results (at baseline and after vaccination). None of them showed platelet-activating properties (Figure 3D–F) compared to a diagnosed HIT patient (Figure 4A) and a VITT patient (Figure 4B).

**Table 3:** Clinical characteristics of patients with thrombotic event following COVID-19 vaccination.

Clinical characteristics	All vaccines	ChAdOx1 nCoV-19	BNT162b2	mRNA 1273
Patients, *n* (%)	32 (100.0)	11 (35.5)	19 (61.3)	1 (3.23)
Clinical characteristics				
Women, *n* (%)	16 (50.0)	6 (54.5)	9 (47.4)	1 (100.0)
Age, (y), median (IQR)	61.0 (47.0-72.8)	64.0 (57.0-73)	54.0 (37.0-73.0)	54.0
Delay between vaccination and thrombosis (d), median (IQR)	11.0 (6.3-19.8)	13.0 (10.0-26.0)	11.0 (7.0-20.0)	1.00
Most common location of thrombosis				
PE with or without DVT	10 (31.3)	3 (27.3)	9 (47.4)	0 (0.0)
Isolated DVT in upper or lower limbs	13 (40.6)	5 (45.5)	6 (31.6)	0 (0.0)
Cerebral venous sinus thrombosis	0 (0.0)	0 (0.0)	0 (0.0)	0 (0.0)
Splanchnic (portal mesenteric) vein thrombosis only	2 (6.3)	0 (0.0)	2 (10.5)	0 (0.0)
Acute coronary syndrome	2 (6.3)	0 (0.0)	1 (5.6)	1 (100.0)
Stroke	1 (3.1)	1 (9.1)	0 (0.0)	0 (0.0)
Obliterative arteriopathy of the lower limbs	1 (3.1)	0 (0.0)	1 (5.6)	0 (0.0)
Splenic infarction	1 (3.1)	1 (9.1)	0 (0.0)	0 (0.0)
Renal artery infarction	1 (3.1)	0 (0.0)	1 (5.6)	0 (0.0)
Time between thrombosis and blood sampling (d), median (IQR)	5.0 (1.0-30.0)	18 (0.0-58.0)	3.0 (1.00-11.0)	6.0
Laboratory tests, *n* (%)				
Platelet count <100 × 10^9^/L	0 (0.0)	0 (0.0)	0 (0.0)	0 (0.0)
Positive IgG anti-PF4/H antibodies	1 (3.1)	0 (0.0)	1 (5.3)	0 (0.0)

Anti-PF4/H, heparin-platelet factor 4 complex antibodies; DVT, deep venous thrombosis; IgG, immunoglobulin G; PE, pulmonary embolism.

### 3.4 Relationship between anti-PF4/H levels and platelet activation in HIT and VITT

**Figure 5:**
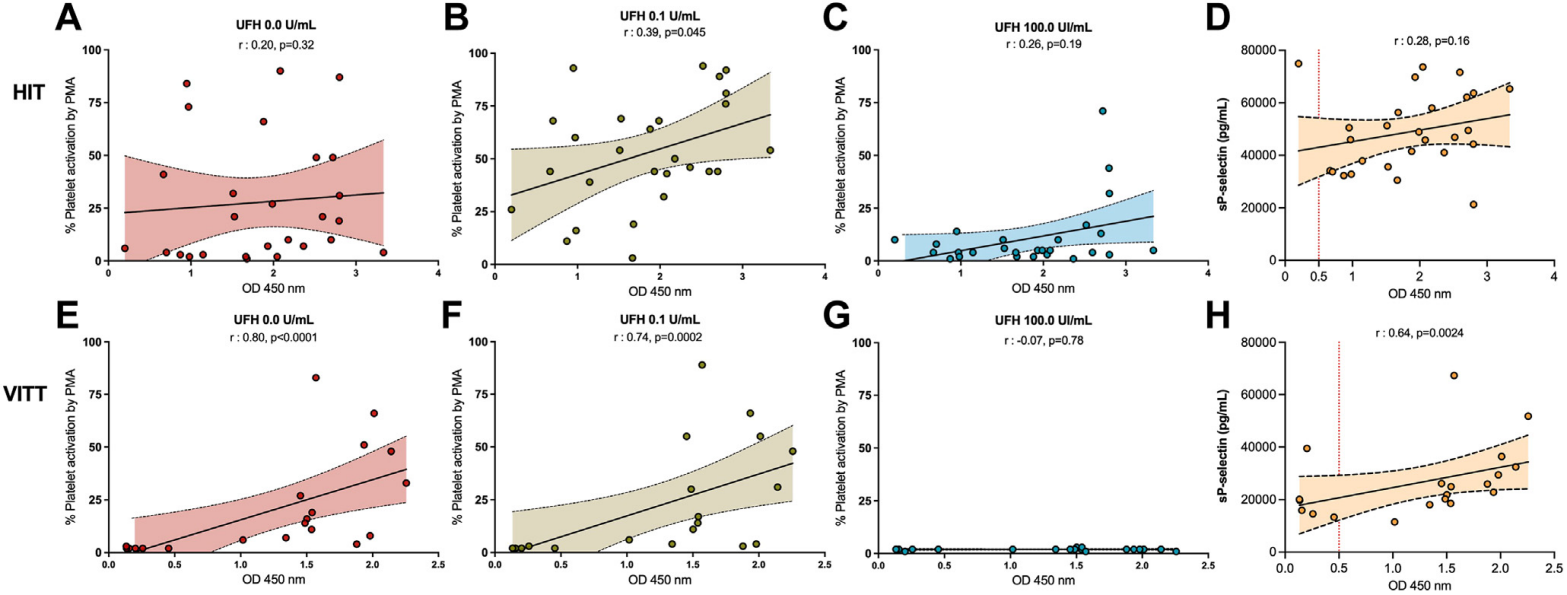
Different platelet activation profiles between patients with confirmed heparin-induced thrombocytopenia (HIT) and with vaccine-induced immune thrombotic thrombocytopenia (VITT). (A) Correlation between the index of platelet activation by flow cytometry (FC) of platelet microvesicle assay (PMA) in buffer (unfractionated heparin [UFH], 0.0 U/mL) and immunoglobulin G (IgG) heparin–platelet factor 4 complex antibodies (anti-PF4/H) levels in samples from confirmed heparin-induced thrombocytopenia (HIT) patients. (B) Correlation between the index of platelet activation by FC of PMA with UFH 0.1 U/mL and IgG anti-PF4/H levels in samples from confirmed HIT patients. (C) Correlation between the index of platelet activation by FC of PMA with UFH 100.0 U/mL and IgG anti-PF4/H levels in samples from confirmed HIT patients. (D) Correlation between soluble P-selectin (sP-selectin) and IgG anti-PF4/H levels in samples from confirmed HIT patients. (E) Correlation between the index of platelet activation by FC of PMA in buffer (UFH 0.0 U/mL) and IgG anti-PF4/H levels in samples from VITT patients. (F) Correlation between the index of platelet activation by FC of PMA with UFH 0.1 U/mL and IgG anti-PF4/H levels in samples from VITT patients. (G) Correlation between the index of platelet activation by FC of PMA with UFH 100.0 U/mL and IgG anti-PF4/H levels in samples from VITT patients. (H) Correlation between sP-selectin and IgG anti-PF4/H levels in samples from VITT patients. OD, optical density.

Next, platelet activation related to anti-PF4/H levels was evaluated in samples from patients with confirmed HIT (*n* = 27 samples at diagnosis) and confirmed VITT (*n* = 20 samples). For VITT patients, samples were collected only at diagnosis for 2 cases, and at both diagnosis and during follow-up for the other 2 cases (Supplementary Figure S1). Washed platelets from healthy donors were tested for activation using PMA, in the presence of patient serum or plasma, and with heparin or PF4 at various concentrations [10,32]. We confirmed, as illustrated in the previous figures, a classical profile of platelet activation that was inhibited by a supratherapeutic dose of UFH in HIT patients (data not shown). Concerning the platelet activation profile in VITT patients, the percentage of platelet activation was not significantly different at the prophylactic dose of UFH compared to buffer but was inhibited at the supratherapeutic dose of UFH or with the use of anti-FcγRIIA (Figure 4C). Furthermore, increased platelet activation in the presence of the presence PF4 was abolished with the use of anti-FcγRIIA in VITT patients. In HIT patients, as expected, no correlation was found between anti-PF4/H levels and platelet activation evaluated by PMA in the absence of heparin (r = 0.20, *P* = .32, Figure 5A) or with a supratherapeutic dose of UFH (r = 0.28, *P* = .19, Figure 5C). However, anti-PF4/H levels and PMA were significantly correlated in the presence of 0.1 U/mL UFH in plasma (r = 0.39, *P* = .045, Figure 5B). Moreover, sP-sel and anti-PF4/H levels in HIT were not significantly correlated (Figure 5D).

In VITT patients, anti-PF4/H levels exhibited a strong association with PMA both in the absence of heparin (r = 0.80, *P* = .0001, Figure 5E) and in the presence of 0.1 U/mL UFH (r = .74, *P* = .0002, Figure 5F). Furthermore, with a supratherapeutic dose of UFH, the percentage of platelet activation was very low and not associated with anti-PF4/H levels (r = −0.007, *P* = .78, Figure 5F). In contrast to HIT patients, sP-sel and anti-PF4/H levels in VITT were significantly correlated (Figure 5H).

## 4 Discussion

This study shows no increase in anti-PF4/H antibodies in COVID-19 or after vaccination, including SID patients. Strikingly, in VITT patients, these antibodies are associated with PMA and sP-sel levels, distinguishing them from HIT patients. This finding suggests that anti-PF4/H antibodies play a different role in the pathophysiology of VITT; however, their relevance outside a clear setting of thrombocytopenia and thrombosis is very poor.

In this study, we investigated IgG anti-PF4/H levels in several populations including i) a cohort of control patients, from before 2010; ii) 2 French cohorts of control individuals included in 2020 and 2021 before and after COVID-19 vaccination at various sampling times, and using different COVID-19 vaccines; iii) a cohort of patients with SID before and after COVID-19 vaccination [25]; and iv) a cohort of consecutive patients referred to our center and presenting with thrombotic events within 30 days after COVID-19 vaccination (Supplementary Table S8). The findings revealed that the prevalence of positive anti-PF4/H was low in the control (5.7%) or before vaccination (0.0%-3.7%) and in patients with SID (1.6%), which seems not different from the prevalence in the general population (2.2%-4.3%) previously described in the literature [10,32], depending on the ELISA used. To the best of our knowledge, the frequency of positivity of anti-PF4 antibodies following COVID-19 vaccination (ChAdOx1 nCov-19 and BNT162b2) ranges from 1.2% to 6.8%, without VITT [33,34]. In the present study, we found that the prevalence of positive anti-PF4/H after COVID-19 vaccination was also low in controls (0.0%-4.8%) and patients with SID (1.7%-2.0%). Importantly, none of the tested individuals exhibited platelet activation using the platelet functional test as observed in previous studies [33,34], particularly in patients with previous autoimmune disorders. VITT incidence remained very low (1 in 26,000 with ChAdOx1 nCoV-19 and 1 in 533,333 with Ad26.COV2.S) after more than 5.7 billion doses were administered globally (on August 15, 2021) and the beneficial effect of COVID-19 vaccination fully outweighs this rare adverse effect [35].

However, the exact pathogenesis of VITT remains to be elucidated, as VITT-related anti-PF4 antibodies do not cross-react with SARS-CoV-2 spike protein [9]. Therefore, this study demonstrates that SARS-CoV-2 infection does not increase anti-PF4 antibodies and that there is no correlation with COVID-19 severity. Additionally, the few individuals who tested positive for anti-PF4/H antibodies do not exhibit platelet activation, as previously described [36]. Schönborn et al. [37] highlighted that SARS-CoV-2 infection does not restimulate anti-PF4 antibodies in patients with a history of VITT, aligning with the absence of a link between SARS-CoV-2 infection and anti-PF4 antibodies. In our study, despite a 2-fold increase in HIT suspicions by physicians during the first wave of the pandemic, the incidence of HIT diagnoses in COVID-19 patients was comparable to that in non–COVID-19 patients, as previously reported in a meta-analysis [6].

Our study explored the association of anti-PF4/H and *ex vivo* platelet activation in the presence or absence of heparin using 2 different techniques, SRA and PMA. These reference methods in HIT and/or VITT allow for the exploration of platelet activation in the presence or absence of heparin. sP-sel is a well-known marker of *in vivo* platelet activation in blood and in particular in COVID-19 [38,39]. In 2024, Nilius et al. [16] used modern proteomic profiling to improve HIT diagnosis, identifying sP-sel as being more abundant in HIT patients, and confirmed by ELISA. Following the COVID-19 pandemic and after the emergence of VITT, a classification of anti-PF4 antibodies as type 1 (nonpathogenic, non-platelet activating), type 2 (heparin dependent, platelet-activating), and type 3 (heparin-independent, platelet-activating) has been proposed by Warkentin and Greinacher [14]. A key finding of the present study is that type 3 antibody levels correlated both with platelet activation evaluated *ex vivo* by specific platelet activation test with PMA, irrespective of heparin, and with *in vivo* platelet activation evaluated with sP-sel. Neither PMA nor sP-sel correlated with the levels of anti-PF4/H in the absence of thrombocytopenia or true clinical suspicion of HIT or VITT. Additionally, the absence of positive results for anti-PF4/H and/or platelet functional tests in patients without thrombocytopenia but with thrombotic event within 1 month after COVID-19 vaccination confirm that VITT should be suspected only in patients who meet the VITT suspicion criteria as defined by the ISTH [15], thrombocytopenia being a major criterion for VITT suspicion and others PF4-related thrombotic disorders.

Furthermore, our study demonstrated the ability to diagnose type 3 antibodies in VITT using IgG antiPF4/H ELISA Zymutest HIA (Hyphen Biomed). While there may be a need to refine the understanding of VITT with specific biological tests [40], it is crucial for nonspecialized laboratories to be able to detect anti-PF4/H with standard ELISA tests before referring to specialized laboratories equipped to handle appropriate tests for type 3 anti-PF4 antibodies.

We acknowledge several limitations in the present study. First, the study periods in 2019 and 2020 are relatively short, resulting in a small sample size for evaluating HIT suspicions and diagnoses. The 2020 study period corresponds to the first wave of the COVID-19 pandemic and the initial French lockdown, during which a reduction in non–COVID-19 activities and overall surgical activity would have been expected to result in fewer HIT suspicions. However, this was not the case. We observed a high number of HIT suspicions without a corresponding increase in HIT diagnoses, consistent with findings from a previous study [6]. Second, we observed a moderate correlation between anti-PF4/H levels and sP-sel only in VITT patients; however, this included repeated measurements from the same individuals during follow-up for two patients. Nevertheless, cases of VITT are very rare, and samples are difficult to obtain. These results should be confirmed in a larger population of VITT patients. Additionally, the absence of a correlation between anti-PF4/H levels and sP-sel in HIT patients should be confirmed in larger samples to definitively establish evidence of an absence of correlation in this setting. Third, in accordance with French legislation, we acknowledge that the absence of race/ethnicity data is a limitation, as sociocultural factors may influence health outcomes, including thrombotic disorders. This limitation may impact the generalizability of our findings to more diverse populations.

All in all, our study highlights the distinct profiles of anti-PF4/H antibodies and platelet activation in HIT and VITT, demonstrating the irrelevance of systematically testing anti-PF4/H during COVID-19 or after vaccination in healthy subjects or in SID. Anti-PF4/H testing is crucial only in cases where thrombotic PF4-related disorders are suspected, such as HIT (using a probability score) or VITT (with thrombosis and thrombocytopenia), following ISTH guidelines. The prevalence of anti-PF4/H in the general population remains low, around 5%, regardless of vaccination or inflammatory status, but with a significant risk of false positive results, potentially leading to misdiagnosis and potential harmful patient management. The discovery of new PF4-related immune disorders underlying VTE during the pandemic has opened new avenues in thrombosis research, requiring further evaluations of new biological detection methods and clinical diagnostics approach to enhance patient care.

## Appendix

**COVID-HOP Study Group participants:** Laurent Abel (Paris, France), Abiramy Arasaratnam (Paris, France), Olivier Aubert (Paris, France), Frédéric Batteux (Paris, France), Lynda Bensefa-Colas (Paris, France), Laure Berton (Paris, France), Cléo Bourgeois (Paris, France), Vincent Calvez (Paris, France), Emmanuelle Cambau (Paris, France), Karine Champion (Paris, France), Marie Courbebaisse (Paris, France), Constance Delaugerre (Paris, France), Nathalie Demory-Guinet (Paris, France), Diane Descamps (Paris, France), Juliette Djadi-Prat (Paris, France), Aurélie Durel Maurisse (Paris, France), Xavier Duval (Paris, France), Jean-Luc Ecobichon (Paris, France), Mathilde Favreau (Paris, France), Gérard Friedlander (Paris, France), Elisabeth Gabarra (Paris, France), Daniela Geromin (Paris, France), Benoit Girard (Paris, France), Pascal Grange (Paris, France), Clémence Granier (Paris, France), Jean-Sébastien Hulot (Paris, France), Laurence Janot (Paris, France), Valérie Jolaine (Paris, France), Pauline Jouany (Paris, France), Yu Jin Jung (Paris, France), Ouifiya Kafif (Paris, France), Najiby Kassis-Chikhani (Paris, France), Solèn Kerneis (Paris, France), Marie Lachatre (Paris, France), Jean-Marc Lacorte (Paris, France), Odile Launay (Paris, France), David Lebaux (Paris, France), Marianne Leruez-Ville (Paris, France), Luong Liem (Paris, France), Amanda Lopes (Paris, France), Martine Louet (Paris, France), Alexandre Loupy (Paris, France), Estelle Lu (Paris, France), Philippe Manivet (Paris, France), Anne-Geneviève Marcelin (Paris, France), Jean-François Meritet (Paris, France), Margaux Monnet (Paris, France), Thomas Padilla (Paris, France), Béatrice Parfait (Paris, France), Claire Pernin (Paris, France), Bruno Pinna (Paris, France), Dominique Prié (Paris, France), Paule Puymoyen (Paris, France), Hélène Péré (Paris, France), Lluis Quintana-Murcy (Paris, France), Flore Rozenberg (Paris, France), Bénédicte Sawicki (Paris, France), Michaela Semeraro (Paris, France), Damien Sene (Paris, France), Éric Tartour (Paris, France), Sarah Tubiana (Paris, France), Benoit Vedie (Paris, France), David Veyer (Paris, France), Aurélie Vilfaillot (Paris, France).
