# Supplementary material for: Relevance of anti–platelet factor 4/heparin antibodies and platelet activation in systemic inflammatory diseases and thrombosis disorders: insight from the COVID-19 pandemic
Source: Res Pract Thromb Haemost. 2025 Feb 9;9(1):102701. doi: 10.1016/j.rpth.2025.102701 (PMC11929090; doi:10.1016/j.rpth.2025.102701)
Supplement: Supplementary Material [file mmc1.docx]

**Supplemental material for “Relevance of anti-PF4/H antibody and platelet activation in Systemic inflammatory diseases and thrombosis disorders: insight from the COVID-19 pandemic”**

Nicolas GENDRON, Dominique HELLEY, Johannes THALER, Dorothée FAILLE, Christine LE BELLER, Maxime GRUEST, Jérôme Hadjadj, Aurélien PHILIPPE, Faris ZECO, Marie COURBEBAISSE, Luc DARNIGE, Wafa AMARA, Leyla CALMETTE, Beatrice PARFAIT, Claire AUDITEAU, Richard CHOCRON, Lina KHIDER, Laetitia MAUGE, Olivier ESPITIA, Gérard FRIEDLANDER, Nadine AJZENBERG, David LEBEAUX, Benjamin PLANQUETTE, Olivier SANCHEZ, Jean-Luc DIEHL, COVID-HOP study group, Agnès LILLO-LE LOUET, Benjamin TERRIER and David M. SMADJA.

**Supplemental Table 1 – Clinical and Biological Characteristics of COVID-19 Patients at Admission and Outcomes in the cohort with anti-PF4/H measured at Admission.**

Anti-PF4/H: anti-platelet factor 4/heparin antibodies; BMI: body mass index; IQR: interquartile range; CRP: C-reactive protein; ICU: intensive care unit.

*Obesity was defined as BMI > 30 kg/m2

|  | **COVID-19 patients**  **(n=81)** |
| --- | --- |
| Male sex – n (%) | 56 (69.1) |
| Age – years, median [IQR] | 66.0 [56.5–73.0] |
| BMI – Kg/m², median [IQR] | 26.6 [24.3–29.4] |
| Time from illness onset to hospital admission - – days, median [IQR] | 7.0 [5.0–11.0] |
| **Comorbidities** |  |
| Obesity*– n (%) | 31 (38.3) |
| Hypertension – n (%) | 43 (53.1) |
| Hyperlipidemia – n (%) | 23 (28.4) |
| Diabetes – n (%) | 26 (32.1) |
| **Biological parameters** | |
| CRP – mg/L, median [IQR] | 88.2 [50.1–151.4] |
| Plasma creatinine – μmol/L, median [IQR] | 81.0 [67.5–106.5] |
| Hemoglobin – g/L, median [IQR] | 132.0 [114.0–144.5] |
| Platelet count – x10^9^ per L, median [IQR] | 215.0 [149.0–281.0] |
| White blood cells – x10^9^ per L, median [IQR] | 6.1 [4.7–7.9] |
| Neutrophils – x10^9^ per L, median [IQR] | 4.6 [3.4–6.5] |
| Monocytes – x10^9^ per L, median [IQR] | 0.4 [0.3–0.6] |
| Lymphocytes – x10^9^ per L, median [IQR] | 0.8 [0.6–1.2] |
| Fibrinogen – g/L, median [IQR] | 5.9 [5.1–6.7] |
| D-dimer – ng/mL, median [IQR] | 1431.0 [854.0–2530.0] |
| Anti-PF4/H– Optical density, median [IQR] | 0.048 [0.034 –0.078] |
| **Outcomes** |  |
| Medicine ward during the whole period of hospitalization – n (%) | 39 (48.1) |
| ICU at admission – n (%) | 27 (33.3) |
| Medicine ward at admission then ICU – n (%) | 15 (18.5) |
| Mechanical ventilation – n (%) | 29 (35.8) |
| In-hospital mortality – n (%) | 16 (19.8) |
| Length of hospitalization – days, median [IQR] | 9.0 [6.0–20.5] |

**Supplemental Table 2 – Clinical and Biological Characteristics of COVID-19 Patients at Admission and Outcomes in the Cohort with anti-PF4/H measured at T0 and T1**

BMI: body mass index; IQR: interquartile range; CRP: C-reactive protein.

*Obesity was defined as BMI > 30 kg/m2

|  | **COVID-19 patients**  **(n=38)** |
| --- | --- |
| Male sex – n (%) | 38 (73.7) |
| Age – years, median [IQR] | 66.0 [58.2–72.3] |
| BMI – Kg/m², median [IQR] | 27.0 [24.7–28.6] |
| **Comorbidities** |  |
| Obesity*– n (%) | 7 (18.4) |
| Hypertension – n (%) | 12 (31.6) |
| Hyperlipidemia – n (%) | 13 (34.2) |
| Diabetes – n (%) | 7 (18.4) |
| **Biological parameters** | |
| CRP – mg/L, median [IQR] | 194.4 [125.9–251.8] |
| Plasma creatinine – μmol/L, median [IQR] | 86.5 [60.8–192.7] |
| Hemoglobin – g/L, median [IQR] | 130.0 [117.0–138.8] |
| Platelet count – x10^9^ per L, median [IQR] | 243.5 [171.8–260.5] |
| White blood cells – x10^9^ per L, median [IQR] | 8.1 [6.0–11.7] |
| Neutrophils – x10^9^ per L, median [IQR] | 6.3 [4.8–11.0] |
| Monocytes – x10^9^ per L, median [IQR] | 0.4 [0.3–0.9] |
| Lymphocytes – x10^9^ per L, median [IQR] | 0.7 [0.5–1.0] |
| Fibrinogen – g/L, median [IQR] | 6.7 [5.9–7.4] |
| D-dimer – ng/mL, median [IQR] | 1423.0 [853.0–2378.0] |
| **Outcomes** |  |
| **Time from admission to intubation** – day, median [IQR] | 2.0 [1.0–4.0] |
| In-hospital mortality – n (%) | 17 (44.7) |

**Supplemental Table 3 – PMA and SRA results from HIT suspected patients in the 2019 and 2020 periods.**

Anti-PF4/H: anti-platelet factor 4-heparin antibodies; PMA: platelet microvesicles assay; SRA: serotonin release assay; UFH: unfractionated heparin.

*HIT diagnosed patient according to functional test results and platelet evolution.

|  | | | | | **PMA (% platelet activation)** | | | | | **SRA (% platelet activation)** | | | | |
| --- | --- | --- | --- | --- | --- | --- | --- | --- | --- | --- | --- | --- | --- | --- |
|  |  |  |  |  | **UFH in U/mL** | | | | | **UFH in U/mL** | | | | |
| **Patient** | **Study period** | **COVID-19**  **statut** | **Score 4T** | **Anti-PF4/H** | **0.0** | **0.1** | **0.5** | **100.0** | **0.1 + anti-FcγRIIa** | **0.0** | **0.1** | **0.5** | **100.0** | **0.1 + anti-FcγRIIa** |
| **1*** | **2019** | Negative | 6 | 3.01 |  |  |  |  |  | 59 | 102 | 119 | 76 |  |
| **2** |  | Negative | 2 | 0.61 | 2 | 2 | 4 | 6 |  | 1 | 5 | 4 | 1 |  |
| **3*** |  | Negative | 4 | 2.59 | 21 | 44 | 52 | 4 |  | 58 | 90 | 89 | 0 |  |
| **4** |  | Negative | 4 | 1.77 | 2 | 2 | 3 | 4 |  | 0 | 0 | 0 | 0 |  |
| **5** |  | Negative | 4 | 0.55 | 5 | 4 | 6 | 8 |  | 1 | 0 | 6 | 6 |  |
| **6*** | **2020** | Negative | 4 | 1.43 | 4 | 17 | 14 | 4 |  | 71 | 80 | 55 | 4 |  |
| **7*** |  | Negative | 4 | 3.25 | 2 | 28 | 40 | 9 |  | 63 | 101 | 101 | 23 |  |
| **8*** |  | Negative | 4 | 2.81 | 11 | 50 | 51 | 9 |  | 80 | 102 | 105 | 45 |  |
| **9*** |  | Negative | 4 | 3.34 | 3 | 16 | 26 | 4 |  | 91 | 89 | 89 | 1 |  |
| **10** |  | Positive | 3 | 0.82 | 76 | 3 | 4 | 3 |  | 50 | 2 | 3 | 0 |  |
| **11** |  | Positive | 5 | 0.72 |  |  |  |  |  | 18 | 85 | 97 | 0 | 7 |
| **12** |  | Positive | 4 | 1.08 | 3 | 3 | 2 | 3 |  | 0 | 6 | 0 | 0 |  |
| **13** |  | Positive | 7 | 3.44 | 46 | 54 | 51 | 34 | 6 | 97 | 96 | 98 | 88 |  |

**Supplemental Table 4 – Clinical and biological characteristic of HIT-suspected patients during 2020-period study**.

*Creatinine clearance was calculated using the Cockcroft and Gault formula.

HIT: heparin-induced thrombocytopenia; IQR: interquartile range; ECMO: extracorporeal membrane oxygenation; UFH: unfractionated heparin; LMWH: Low-molecular-weight heparin; SD: standard deviation.

| **HIT-suspected patients in 2020-group** | **Non-COVID-19 patients (N=19)** | **COVID-19 patients (N=23)** | **p-value** |
| --- | --- | --- | --- |
| **Age - years, median [IQR]** | 63.0 [52.5–72.5] | 66.0 [60.0–79.5] | 0.32 |
| **Male sex – n (%)** | 12 (63.2) | 19 (82.6) | 0.28 |
| **BMI – kg/m², median [IQR]** | 23.6 [20.4–29.5] | 26.3 [24.9–27.8] | 0.41 |
| **Medicine ward** | 4 (21.1) | 6 (26.1) | NA |
| **Intensive care** | 9 (47.4) | 16 (69.6) | NA |
| **Cardiac or orthopedic surgery** | 6 (31.6) | 1 (4.3) | NA |
| **Cardiopulmonary bypass – n (%)** | 5 (26.3) | 0 (0.0) | **0.032** |
| **ECMO – n (%)** | 4 (21.1) | 2 (9.1) | 0.52 |
| **UFH at suspicion – n (%)** | 6 (31.6) | 7 (30.4) | 1.00 |
| **LMWH at suspicion – n (%)** | 13 (68.4) | 16 (69.6) | 1.00 |
| **Duration of heparin exposition before HIT diagnosis, mean (SD)** | 8.5 [6.3–15.0] | 15.0 [8.5–18.5] | 0.06 |
| **Therapeutic anticoagulation – n (%)** | 13 (68.4) | 18 (78.3) | 0.71 |
| **Heparin exposure within 3 months – n (%)** | 0 (0.0) | 1 (4.8) | 1.00 |
| **Platelet count – G/L, mean (SD)** | 76.0 [53.5–105.0] | 86.0 [68.0–128.0] | 0.21 |
| **White blood cells - G/L, mean (SD)** | 12.2 [7.08–15.4] | 12.7 [8.6–17.6] | 0.99 |
| **Hemoglobin - g/L, mean (SD)** | 88.5 [82.0–99.3] | 88.0 [80.0–99.0] | 0.82 |
| **PT ratio- %, mean (SD)** | 66.0 [62.0–74.0] | 72.0 [63.3–77.8] | 0.52 |
| **Fibrinogen - g/L, mean (SD)** | 5.2 [3.8–7.0] | 6.0 [4.6–8.2] | 0.26 |
| **Factor V - %, mean (SD)** | 97.0 [91.0–111.0] | 134.0 [95.0–140.0] | 0.20 |
| **D-dimer - ng/mL, mean (SD)** | 2731 [1984–7396] | 3660 [2997–5977] | 0.66 |
| **Creatinine clearance* - ml/min, mean (SD)** | 45.2 [35.9–108.7] | 46.9 [25.4–104.9] | 0.96 |
| **Nadir hemoglobin - g/L, mean (SD)** | 80.0 [73.0–89.0] | 71.0 [67.0–85.0] | 0.17 |
| **Nadir platelets - G/L, mean (SD)** | 85.0 [46.0–105.0] | 62.5 [40.0–107.3] | 0.71 |
| **Venous thrombosis – n (%)** | 3 (16.7) | 3 (13.6) | 1.00 |
| **Arterial thrombosis – n (%)** | 3 (15.8) | 3 (13.0) | 1.00 |

**Supplemental Table 5 – Demography and clinical characteristics of controls enrolled in the FARIVE study.**

IQR: interquartile range.

*Obesity was defined as BMI > 30 kg/m2

| **Controls_2003-2010_ (n=122)** | |
| --- | --- |
| **Clinical characteristics** | |
| Women – n (%) | 80 (65.6) |
| Age – years, median (IQR) | 53.0 (38.5–67.0) |
| Body mass index – Kg/m^2^ (IQR) | 24.3 (21.6–28.2) |
| Blood group – n (%) |  |
| A | 53 (43.4) |
| B | 6 (4.9) |
| AB | 4 (3.3) |
| O | 50 (41.0) |
| History of cancer | 5 (4.1) |
| History of surgery | 108 (88.5) |
| **Comorbidities** |  |
| Obesity*– n (%) | 24 (19.7) |
| Hypertension – n (%) | 45 (36.9) |
| Hyperlipidemia – n (%) | 26 (21.3) |
| Diabetes – n (%) | 15 (12.3) |

**Supplemental Table 6 – Demography and clinical characteristics of controls enrolled in the COVADIS study**

IQR: interquartile range.

| **Controls_2021_ (n=24)** | |
| --- | --- |
| **Clinical characteristics** | |
| Women - n (%) | 8 (33.3) |
| Age - years, median (IQR) | 50.0 (32.5–59.0) |
| **COVID-19 vaccine** - n (%) |  |
| BNT162b2 | 22 (91.7) |
| ChAdOx1 nCoV-19 | 2 (8.3) |

**Supplemental Table 7 – Demography and clinical characteristics of healthcare workers controls enrolled in the COVID-HOP study**

IQR: interquartile range.

*confirmed with SARS-CoV-2 RT-PCR testing

**Among them, 4 controls received their first vaccine dose with ChAdOx1 nCoV-19* and their second dose with BNT162b2.

| **Controls_HCW_ n=182** | |
| --- | --- |
| **Clinical characteristics** | |
| Women - n (%) | 148 (81.3) |
| Age - years, median (IQR) | 47.0 (37.0–54.3) |
| Body mass index – Kg/m^2^ (IQR) | 24.3 (21.2–27.7) |
| Blood group - n (%) |  |
| A | 64 (35.6) |
| B | 17 (9.3) |
| AB | 10 (5.5) |
| O | 66 (36.3) |
| **Previous SARS-CoV-2 infection* - n (%)** |  |
| Yes | 48 (26.4) |
| No | 57 (31.3) |
| Non-available | 77 (42.3) |
| **COVID-19 vaccine** - n (%) |  |
| BNT162b2** | 147 (80.8) |
| ChAdOx1 nCoV-19** | 37 (20.3) |
| mRNA-1273 | 2 (1.1) |

**Supplemental Table 8 – Summary of suspicions and diagnoses of Heparin-Induced Thrombocytopenia and Vaccine-Induced Immune Thrombotic Thrombocytopenia in all patients included in the different cohorts studied.**

HIT: heparin-induced thrombocytopenia; VITT: vaccine-induced immune thrombotic thrombocytopenia; TE: thrombotic event; HCW: healthcare worker.

| **Cohort studied** | **Study** | **Number of included Patients** | **HIT suspicion - n (%)** | **Confirmed HIT - n (%)** | **VITT suspicion - n (%)** | **Confirmed VITT - n (%)** |
| --- | --- | --- | --- | --- | --- | --- |
| **COVID-19 patients with sampling at admission** | SARCODO 2020-A01048-31A, NCT04624997 | 81 | 0 (0.0) | 0 (0.0) | 0 (0.0) | 0 (0.0) |
| **COVID-19 patients at sampling at T0 and T1** |  | 38 | 0 (0.0) | 0 (0.0) | 0 (0.0) | 0 (0.0) |
| **Patients with HIT suspicion during 2019 (March 15 to May 15)** | RESTI-HOP study (N° 2022-03-15, CERAPHP.5, IRB registration: #00011928). | 17 | 17 (100.0) | 2 (11.8) | 0 (0.0) | 0 (0.0) |
| **Patients with HIT suspicion during 2020 (March 15 to May 15)** |  | 42 | 42 (100.0) | 4 (9.5) | 0 (0.0) | 0 (0.0) |
| **Controls_2003-2010_** | FARIVE study (Paris Ile‐de‐France Broussais 2002‐11‐26, FARIVE) | 122 | 0 (0.0) | 0 (0.0) | 0 (0.0) | 0 (0.0) |
| **Controls_2021_** | COVADIS study | 24 | 0 (0.0) | 0 (0.0) | 0 (0.0) | 0 (0.0) |
| **Patients with systemic inflammatory diseases** |  |  |  |  |  |  |
| **Controls_HCW_** | COVID-HOP study (NCT04418375) | 182 | 0 (0.0) | 0 (0.0) | 0 (0.0) | 0 (0.0) |
| **Patients with TE following vaccination** | VITT study (protocol # 20210917154757, CERAPHP.5, IRB registration: #00011928) | 32 | 0 (0.0) | 0 (0.0) | 32 (100.0) | 0 (0.0) |
| **HIT patients** | RESTI-HOP study (N° 2022-03-15, CERAPHP.5, IRB registration: #00011928). | 27 | 27 (100.0) | 27 (100.0) | 0 (0.0) | 0 (0.0) |
| **VITT patients** | EVTF-act study (EK 404-2009) | 4 | 0 (0.0) | 0 (0.0) | 4 (100.0) | 4 (100.0) |

**Supplemental Figure 1 – Platelet Activation by flow cytometry of platelet microvesicle assay and IgG anti-PF4/H levels in two VITT confirmed patients with samples collected at diagnosis and follow-up.**

VITT: vaccine-induced immune thrombotic thrombocytopenia; UFH: unfractionated heparin; PF4: platelet factor 4; OD: optical density.

**
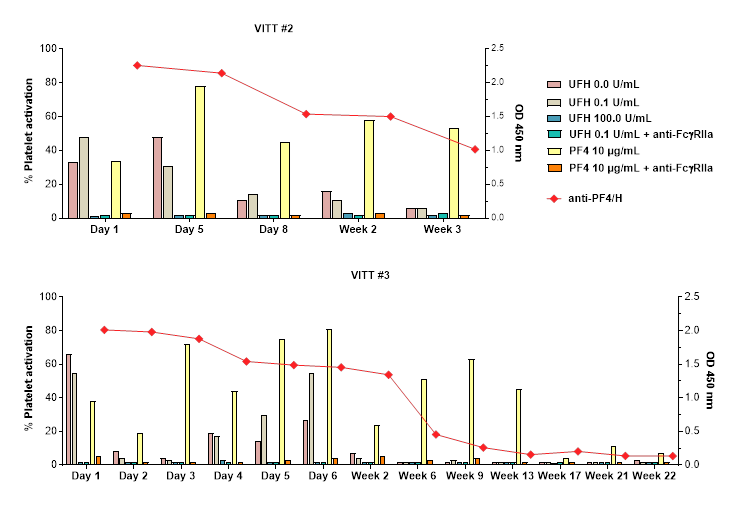
**

**COVID-HOP study group**

**Coordinating Investigator:** Prof. Marie COURBEBAISSE, Physiology Department, European Hospital Georges-Pompidou (HEGP), Assistance Publique – Hôpitaux de Pairs (AP-HP)

**Biological Resources Center (CRB) Coordination:** Dr. Béatrice PARFAIT, Head of CRB at Cochin hospital

**Clinical Research Unit:** Juliette DJADI-PRAT, Pauline JOUANY, and Cléo BOURGEOIS, Estelle LU, Aurélie VILFAILLOT, Abiramy ARASARATNAM HEGP, AP-HP

**Chair of the Scientific Committee:** Prof. David LEBEAUX, Microbiology Department, HEGP, AP-HP

**Scientific Committee:**

- Dr. Laurent ABEL, Human Genetics of Infectious Diseases Laboratory, University of Paris/INSERM U.1163, Imagine Institute, Necker-Enfants Malades Hospital, AP-HP
- Prof. Frédéric BATTEUX, Immunology Department, Cochin Hospital, AP-HP
- Dr. Lynda BENSEFA-COLAS, Coordinator Central Occupational Health Service, AP-HP
- Prof. Vincent CALVEZ, Virology Department, La Pitié-Salpêtrière Hospital, AP-HP
- Prof. Xavier DUVAL, Clinical Investigation Center, Bichat Hospital, AP-HP
- Prof. Gérard FRIEDLANDER, Dean of the Faculty of Medicine, Paris Cité University
- Prof. Jean-Sébastien HULOT, Clinical Investigation Center, HEGP, AP-HP
- Dr. Najiby KASSIS-CHIKHANI, Hospital Hygiene Department, HEGP, AP-HP
- Dr. Solèn KERNEIS, Infectiologist, Cochin Hospital, AP-HP
- Prof. Odile LAUNAY, Clinical Investigation Center in Vaccinology, Cochin Hospital, AP-HP
- Prof. Alexandre LOUPY and Dr. Olivier AUBERT, Adult Renal Transplantation Nephrology Department, Necker-Enfants Malades Hospital, AP-HP
- Dr. Béatrice PARFAIT, CRB Cochin Hospital, AP-HP
- Dr. Hélène PÉRÉ and Dr. David VEYER, Virology Department, HEGP, AP-HP
- Prof. Dominique PRIÉ, Medical Director of the University Medical Department of Medical Biology, Genomic Medicine, Physiology, unit: EOH, AP-HP
- Dr. Lluis QUINTANA-MURCY, Pasteur Institute (Unit of Human Evolutionary Genetics, CNRS UMR2000) & Collège de France (Chair of Human Genomics and Evolution)
- Prof. Éric TARTOUR and Dr. Clémence GRANIER, Immunology Department, HEGP, AP-HP

Hospitals from Assistance Publique Hôpitaux de Paris, Paris. France.

**Investigator (by center):**

- **Lariboisière:** Damien Sene, Karine Champion, Amanda Lopes, Margaux Monnet, Laure Berton
- **Cochin and Hôtel-Dieu:** Marie Lachatre ; Luong Liem, Belarbi Linda, Pascal Grange ; Aurélie Durel Maurisse, Yu Jin Jung, Paule Puymoyen, Mathilde Favreau
- **Necker–Enfants Malades:** Michaela Semeraro, Valérie Jolaine,
- **Hôpital européen Georges Pompidou**: Nathalie Demory-Guinet, Laurence Janot,
- **Pitié-Salpêtrière:** Bruno Pinna, Martine Louet
- **Bichat-Claude Bernard:** Bénédicte Sawicki, Jean-Luc Ecobichon,
- **Corentin Celton:** Elisabeth Gabarra

**Virologist (by center):**

- **Cochin and Hôtel-Dieu:** Flore Rozenberg and Jean-François Meritet
- **Pitié-Salpêtrière:** Anne-Geneviève Marcelin, Vincent Calvez
- **Necker–Enfants Malades:** Marianne Leruez-Ville
- **Bichat-Claude Bernard:** Diane Descamps, Emmanuelle Cambau
- **Lariboisière:** Constance Delaugerre
- **Hôpital européen Georges Pompidou** : Hélène Péré, David Veyer

**Biological Resources Center (by center):**

- **Cochin and Hôtel-Dieu:** Beatrice Parfait, Benoit Girard,
- **Pitié-Salpêtrière:** Jean-Marc Lacorte, Thomas Padilla,
- **Necker–Enfants Malades:** Marianne Leruez-Ville
- **Bichat-Claude Bernard:** Sarah Tubiana, Ouifiya Kafif
- **Lariboisière:** Philippe Manivet, Claire Pernin.
- **Hôpital européen Georges Pompidou**: Benoit Vedie, Daniela Geromin
